# Disentangling the Origins of Cultivated Sweet Potato (*Ipomoea batatas* (L.) Lam.)

**DOI:** 10.1371/journal.pone.0062707

**Published:** 2013-05-27

**Authors:** Caroline Roullier, Anne Duputié, Paul Wennekes, Laure Benoit, Víctor Manuel Fernández Bringas, Genoveva Rossel, David Tay, Doyle McKey, Vincent Lebot

**Affiliations:** 1 Unité Mixte de Recherche Amélioration et Adaptation des plantes (UMR AGAP), Centre International de Recherches en Agronomie pour le Développement (CIRAD), Montpellier, France; 2 Centre d'Ecologie Fonctionnelle et Evolutive (CEFE), Centre National de la Recherche Scientifique (CNRS), Montpellier, France; 3 Université Montpellier II, Montpellier, France; 4 International Potato Center (CIP), Lima, Peru; 5 Institut Universitaire de France (IUF), Paris, France; Montreal Botanical Garden, Canada

## Abstract

Sweet potato (*Ipomoea batatas* (L.) Lam., Convolvulaceae) counts among the most widely cultivated staple crops worldwide, yet the origins of its domestication remain unclear. This hexaploid species could have had either an autopolyploid origin, from the diploid *I. trifida*, or an allopolyploid origin, involving genomes of *I. trifida* and *I. triloba*. We generated molecular genetic data for a broad sample of cultivated sweet potatoes and its diploid and polyploid wild relatives, for noncoding chloroplast and nuclear ITS sequences, and nuclear SSRs. Our data did not support an allopolyploid origin for *I. batatas*, nor any contribution of *I. triloba* in the genome of domesticated sweet potato. *I. trifida* and *I. batatas* are closely related although they do not share haplotypes. Our data support an autopolyploid origin of sweet potato from the ancestor it shares with *I. trifida,* which might be similar to currently observed tetraploid wild *Ipomoea* accessions. Two *I. batatas* chloroplast lineages were identified. They show more divergence with each other than either does with *I. trifida*. We thus propose that cultivated *I. batatas* have multiple origins, and evolved from at least two distinct autopolyploidization events in polymorphic wild populations of a single progenitor species. Secondary contact between sweet potatoes domesticated in Central America and in South America, from differentiated wild *I. batatas* populations, would have led to the introgression of chloroplast haplotypes of each lineage into nuclear backgrounds of the other, and to a reduced divergence between nuclear gene pools as compared with chloroplast haplotypes.

## Introduction

Polyploidy is recognized as an important factor in the evolution and diversification of plants [1]. Polyploid crops are common, and include for example banana, bread wheat, potato, sugar beet and sweet potato, and polyploidy is frequently used by breeders for crop improvement. Crop domestication corresponds to an evolutionary process of species divergence, in which genetic, morphological and physiological changes result from the cultivation of plants by humans [2]. Often considered an “event”, particularly for clonally propagated crops [3], domestication is increasingly looked upon as a protracted process, involving repeated recombination-selection cycles and often wild/cultivated gene flow, with artificial (conscious or not) and natural selection interacting to drive the wild-to-domesticated transition [4], [5]. The link between polyploidy and domestication is not clearly established, although some have speculated that polyploidy may predispose crops for domestication [6].

In natural populations, polyploid species may be formed through several mechanisms. Classically, autopolyploidy (genome duplication with a single progenitor species) has been distinguished from allopolyploidy (hybridization and genome doubling of highly divergent parental species; [1]). However, there is a continuum between the two. Autopolyploid complexes often have multiple independent origins, sometimes involving crosses between conspecific, but still substantially differentiated, populations [7]. Polyploidization often triggers genomic re-patterning and gene expression changes [1], which could explain the sudden appearance of new phenotypes that diverge from those of their diploid parents in numerous traits. Although these genetic changes are probably more rapid and extensive in allopolyploids, they may also affect autopolyploids over the longer term [7]. Moreover, polyploids may be reproductively isolated from their parents, and often can adapt to new ecological niches [1]. Shifts towards higher ploidy levels thus often drive speciation in plants [8], and indeed appear as a clear route to sympatric speciation [9]. In this context, autopolyploidy seems to have a higher incidence than previously assumed [7]. While the proportion of polyploids among crops is not statistically different from that among wild species of the same families [10], in some cases, polyploidy certainly provided raw material to achieve plant domestication. For example, the exploitation of fertile diploid banana genotypes began in New Guinea in the early Holocene. Human-mediated transfers of these diploids through islands of Melanesia and South-Eastern Asia have allowed hybridizations between allopatric subspecies of diploid *Musa acuminata*. As an evolutionary consequence of this hybrid status, seedless triploid genotypes were formed and widely cultivated [11].

Numerous crops were domesticated in the Americas, including potato, tomato, manioc, maize, beans, sweet potato, squash and many others [12]. Even though some crops were domesticated only once, in a restricted area (as is the case for maize; [13], [14]), most of them were domesticated over a diffuse area [15], and some were domesticated two or more times independently. Common bean [16] and Lima bean [17] were domesticated several times each, in different parts of the range of their respective ancestral species, and the domesticated gene pools came into contact only secondarily. Whether a particular crop was domesticated once or several times, and from which species, is of more than historical interest. Independent domestication events raise the fundamental issue of determining how multiple genetic paths could lead to similar domestication traits, and documenting independent domestication provides essential data for the management of crop diversity, for the conservation of genetic resources, and for the use of wild relatives in breeding programs.

Sweet potato (*Ipomoea batatas* (L.) Lam., Convolvulaceae) is a clonally propagated hexaploid crop native to the Americas. It is a major staple crop, particularly in numerous tropical countries [18]. Despite its importance, the botanical origin of sweet potato and the timing and geographic location(s) of its domestication remain unclear. Plants classified as *Ipomoea batatas* are mostly cultivated, hexaploid clones. Several molecular-genetic studies appeared to indicate that the diploid *I. trifida* is the closest wild relative of cultivated sweet potato [19]–[24], thus pointing to this species as the most likely candidate progenitor of sweet potato. Two hypotheses have been proposed as to the origin of cultivated sweet potato. On the one hand, Austin [25] proposed that natural hybridization between *I. trifida* and *I. triloba* could have generated the ancestors of sweet potato somewhere between the Yucatan peninsula and the Orinoco basin. On the other hand, Kobayashi [26] proposed that *I. trifida* forms an autopolyploid complex, with ploidy levels ranging from diploid to hexaploid, and that domesticated sweet potato derives from this group.

However, several accessions of *Ipomoea* with various ploidy levels (mostly 4X, but also a few 3X and 6X) are clearly closely related to *I. batatas*, but their taxonomic status is disputed and they are poorly characterized genetically. Collected in Ecuador, Colombia, Guatemala and Mexico, these accessions were initially identified as *I. trifida*, and after re-examination, most of them were re-identified as wild *I. batatas* on taxonomical grounds [27]. Whether these could represent wild *I. batatas*, or feral forms, has not been investigated. However, their discovery strongly suggested the possibility that *I. batatas* includes not only the hexaploid cultigen but also true wild populations with lower ploidy levels, from which cultivated forms would have been domesticated. Whether domestication preceded polyploidization (as in bananas, [11]) or followed it (as in cotton, [28]) is still unknown. Furthermore, recent findings point towards a possible double origin of domestication for sweet potato: cultivated landraces comprise two distinct geographically clustered chloroplast lineages [29], one corresponding mostly to landraces cultivated in Central America and the Caribbean (hereafter termed the Northern lineage), and the other to landraces found in northwestern South America (hereafter Southern lineage). Nuclear microsatellite markers confirmed this differentiation pattern [29]. The Northern and Southern genepools could thus represent two independent polyploidization and domestication histories.

In the present study, we address the issues of the botanical and geographic origin of sweet potato, using a representative sampling of *I. batatas*, its putative close wild diploid relatives *I. trifida* and *I. triloba*, and polyploid *Ipomoea* sp., using several neutral nuclear and chloroplast markers. We pose the following questions: Are *I. batatas* gene pools derived from a single progenitor, from differentiated conspecific populations that hybridized, or from multiple hybridizing species? Can we identify the progenitor(s) and pinpoint the geographical origin(s) of domestication? The formation of sweet potato's hexaploid genome must have involved at least two steps, from diploidy to intermediate ploidy levels (triploid or tetraploid) and then hexaploidy. Can we establish the temporal sequence of domestication relative to polyploidization?

## Materials and Methods

### Sampling

Overall, 297 leaf tissue samples were collected from 219 accessions, representing six species from *Ipomoea* series *Batatas* as well as samples of polyploid accessions of dubious taxonomy (Tables S1 and S2). Accessions are synonymous to clones for the cultivated *I. batatas* and to populations for the wild relatives. Sampling for wild taxa includes 1–11 (median 2) individuals per population.

**Figure 1:**
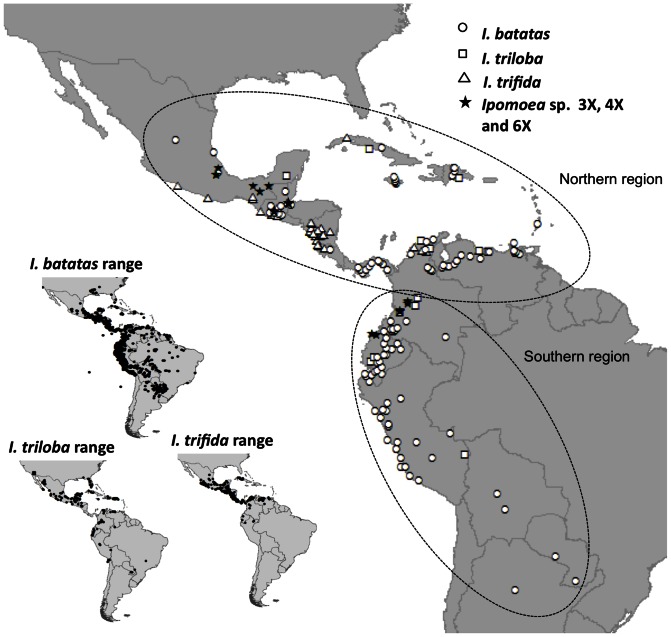
Sampling geographical distribution. Location of *I. triloba*, *I. trifida*, *I. batatas* and polyploid *Ipomoea* sp. accessions used in the present study and current taxon distribution ranges, as determined from GBIF records (http://data.gbif.org/species/) are provided. Accessions with no geographical information are not shown; details on sampling are provided in Tables S1.

*Ipomoea batatas* (139 accessions) was sampled throughout its distributional range from Mexico to Peru, to represent both domesticated gene pools ([29]; 139 accessions). *Ipomoea trifida* (40 accessions; 75 samples; all of them diploid as attested by flow cytometry) and *I. triloba* (15 accessions; 26 samples) were sampled throughout their distributional ranges (Figure 1). One accession of *I. tabascana* was included (2 samples), since this taxon has been postulated to be a hybrid between *I. batatas* and *I. trifida* [30]. Three accessions of *I. leucantha* (6 samples) and four of *I. tiliacea* (9 samples) were sampled as outgroups. Accessions were obtained from the International Potato Center (CIP, Lima, Peru), the National Genetic Resources Program (NGRP, USDA, USA), and the Japanese National Institute for Agrobiological Sciences (NIAS, Tsukuba, Japan) (Tables S1 and S2). Their taxonomical identity corresponds to that mentioned in the *ex-situ* collection passport data. We also recovered in the same collections, samples of uncertain taxonomical status, sometimes referred to in the literature as wild and/or feral *I. batatas* [27], and sometimes as polyploid *I. trifida* [26], [31] (17 accessions; one diploid, one triploid, 13 tetraploid and two hexaploid accessions). Hereafter in this study, these accessions will be termed *Ipomoea* sp. Leaf tissue was collected and dried and DNA was extracted using the Qiagen 96 Plant kit for lyophilised tissues (Hilden, Germany). For some accessions, ploidy level was verified by flow cytometry (Tables S1).

### DNA sequencing

The chloroplast intergenic spacer rpl32-trnL(UAG) [32] and the nuclear ITS region (ITS4-ITS5; [33]) were sequenced for all wild accessions and for a subsample of 23 cultivated *I. batatas* representing both cp lineages as defined in Roullier et al. [29] (164 samples in total; Tables S1 and S2). PCRs were performed in a final volume of 20 μL, using 2 μL of 1:10 diluted template DNA, 0.5 μM of each primer, and 10 μL of Phusion Taq mastermix (New England Biolabs, Inc.). Amplification was performed after 5 minutes denaturation at 98°C, over 30 cycles of 30 s denaturation at 98°C, 1 minute annealing at 57°C and 1 minute elongation at 72°C, and a final elongation of 5 minutes, using a PTC-100 Thermocycler (MJ Research, Waltham, MA, USA). Fluorescent dye-terminator sequencing was performed by Agowa GmbH (Berlin, Germany). All DNA samples were sequenced in forward and backward directions, and ten random duplicates were sequenced for quality control (all duplicates gave congruent results).

### Sequence analysis and phylogenetic reconstruction

Forward and reverse chromatograms were assembled and visually checked independently by two investigators using Sequencher (Gene Codes, Ann Arbor, MI, USA). Only those chromatograms that produced a clear consensus were used in the following analyses. ITS sequences resulted in numerous heterozygotes. Length-variant heterozygotes were systematically discarded, while length-invariant heterozygotes were retained and coded with ambiguous character states when secondary peaks reached 50% of the main one. Sequences were aligned using Muscle (Edgar 2004, using the EMBL web service available at http://www.ebi.ac.uk/Tools/msa/muscle/). The resulting alignment was edited using BioEdit (Hall 1999). All mononucleotide repeats were discarded since these are prone to homoplasy [34], and indels were coded as binary characters, using the simple method of Simmons & Ochoterena [35].

Bayesian and maximum likelihood reconstruction of the phylogenetic tree was performed for plastid data using MrBayes [36] and PhyML 3.0 [37] respectively, using the *I. purpurea* NCBI GenBank database sequence NC_009808.1 (122510–123547 bp) as an outgroup. The most likely model for sequence evolution was selected among those implemented in FindModel (http://www.hiv.lanl.gov/content/sequence/findmodel/findmodel.html) using the Akaike Information Criterion. Since FindModel does not admit binary character coding, model selection did not take indels into account. MrBayes was run four times with four chains, for 10 million iterations. Convergence was attained after 2.5 million iterations, which were discarded as burn-in. A statistical parsimony haplotype network was also constructed for plastid data using TCS 1.21 [38], with indels treated as a fifth character state.

For the ITS sequences, maximum likelihood reconstruction was performed with PhyML 3.0 [37] using the *I. purpurea* NCBI GenBank database sequence AY538318 and the most likely model for sequence evolution as determined in FindModel. As PhyML does not take into account ambiguous characters, we also used the program SPLITSTREE version 4.12.8 [39] to construct a Neighbor-joining tree based on Hamming distance (which handles ambiguous characters). Robustness was assessed through 1,000 bootstrap resamplings. All sequences were deposited in GenBank (Table S1).

### SSR genotyping

283 individuals (belonging to 7 taxa) were genotyped for eight nuclear microsatellites (J263, J522A, Ib297, J206A, IbR16, IbC5, J544b, IbS11) described in a previous study [29]. All loci were amplified independently using Multiplex PCR Taq (Qiagen) in a final volume of 10 µL, using 30 ng of DNA per reaction. The following programme was conducted using a PTC-100 Thermocycler (MJ Research, Waltham, MA, USA): 15 min at 95°C, 35 cycles of 30 s at 94°C, 1 min 30 s at 57°C, 1 min at 72°C and finally 30 min at 72°C. Allele scoring was visually checked by two investigators using GeneMapper (Applied Biosystems, Foster City, CA, USA).

### SSR data analysis

#### Diversity

We characterized the genetic diversity present in geographically well-sampled species (*I. batatas*, *I. trifida*, *I. triloba* and *Ipomoea* sp.), by computing the mean number of alleles per locus (*NA*), its rarefied value *Ar* (averaged from 1000 resamplings of 26 individuals), the observed and expected heterozygosities *Ho* and *He* (the latter was determined for polyploid taxa from allelic frequencies estimated using the R package polysat; “simpleFreq” method), and the intra-taxon mean pairwise Lynch distance between genotypes *D*.

#### Taxon boundaries

The SSR dataset was coded in a binary manner similar to that used for AFLP data. Using the program SPLITSTREE4 version 4.12.8 [39], a NeighborNet was constructed based on a Jaccard distance matrix. Genetic relationships between *I. batatas* and its relatives were further examined by clustering approaches.

We first relied on a non model-based clustering method, the Discriminant Analysis of Principal Components (DAPC), a multivariate analysis implemented in the adegenet R library [40], [41]. The DAPC provides an efficient description of genetic clusters, using a few synthetic functions, called the discriminant functions. This method seeks linear combinations of the original variables (alleles) maximizing between-group differences while minimizing within-group variation. Based on the retained discriminant functions, the analysis derives, for each individual, probabilities of membership in each of the different groups. These coefficients can be interpreted as “genetic proximity” of individuals to the different clusters, and provide an “assignment measure” of individuals to genetic clusters [40], [41]. DAPC in itself requires construction of prior groups [41]. Thus, we first ran a sequential K-means clustering algorithm for K = 2 to K = 10. Using the Bayesian Information Criterion (BIC), we could identify the optimal number of genetic clusters describing the data (in our case, five groups). We then performed DAPC for K = 5, retaining 15 PCA components (the “optimal” value following the a-score optimization procedure proposed in adegenet).

For comparison purpose, we also ran the Bayesian model-based clustering algorithm implemented in the software Structure [42], [43], assuming an admixture model, with allelic frequencies correlated among clusters, and dominant markers coding. 1.5 million MCMC steps were performed, with the first 500,000 iterations discarded as burn-in.

## Results

### Interspecific relationships as inferred from cpDNA sequences

The 1077-bp long alignment of rpl32-trnL(UAG) sequences showed 65 polymorphic sites, 19 of which were parsimony-informative, and 14 indels (once mononucleotide repeats were removed) resulting in 22 haplotypes.

**Figure 2:**
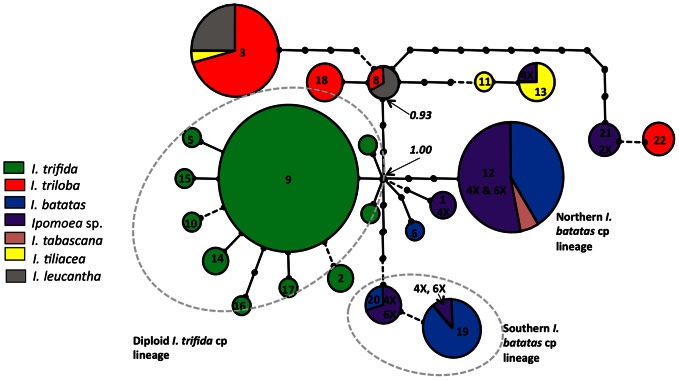
Genetic relationships of *I. batatas*, five wild relatives and *Ipomoea* sp. accessions based on chloroplast DNA analyses. Statistical Parsimony Network of rpl32-trnL(UAG) haplotypes. Circle size is proportional to the number of individuals per haplotype. Substitutions and inversions are represented using full lines and indels are displayed using broken lines. Intermediate, unsampled haplotypes appear as dots. The posterior probability of two nodes, as obtained through a Bayesian tree reconstruction (Figure S1), is reported in italics. The ploidy level of *Ipomoea* sp. accessions is indicated.

Despite extensive geographic sampling of *I. trifida*, *I. triloba* and *I. batatas*, we found no haplotypes shared between any two of these species. *Ipomoea batatas*, *I. trifida* and *I. tabascana* together with the *Ipomoea* sp. polyploid samples form a consistent monophyletic group (Bayesian posterior probability of 1; Figure 2 and Figure S1), but excluding any *I. triloba*.

Out of 72 samples, 61 *I. trifida* shared haplotype 9 and the others carried haplotypes derived from this haplotype by one or two mutation steps (Figure 2). Only four haplotypes were found over the 139 samples of *I. batatas*. As found by Roullier et al. [29], two distinct chloroplast lineages were identified in *I. batatas*, mostly corresponding to Northern and Southern accessions. They were more divergent from each other than each is from *I. trifida* (Figure 2).

The *I. tabascana* sample and numerous samples of uncertain taxonomy (triploid, tetraploid and hexaploid *Ipomoea* sp.) carried the typical Northern *batatas* haplotype, while five tetraploid *Ipomoea* sp. samples carried a Southern *batatas* haplotype, three of them originated from Ecuador and two from Mexico (The unique diploid *Ipomoea* sp. carried a haplotype very close to that borne by one accession labelled as *I. triloba*, but distantly related to other *I. triloba* haplotypes, suggesting they may together form a distinct species. Additionally, one tetraploid *Ipomoea* sp. sample, probably misidentified, bore a haplotype specific to *I. tiliacea)*.

Concerning other species, phylogenetic relationships are less clearly resolved (Figures 2 and S1). Moreover, some haplotypes are shared by accessions identified as different species, suggesting misidentifications or alternatively introgressive hybridization (for example, haplotype 3 is shared among three species, *I. triloba*, *I. leucantha* and *I. tiliacea*).

### Interspecific relationships as inferred from ITS sequences

**Figure 3:**
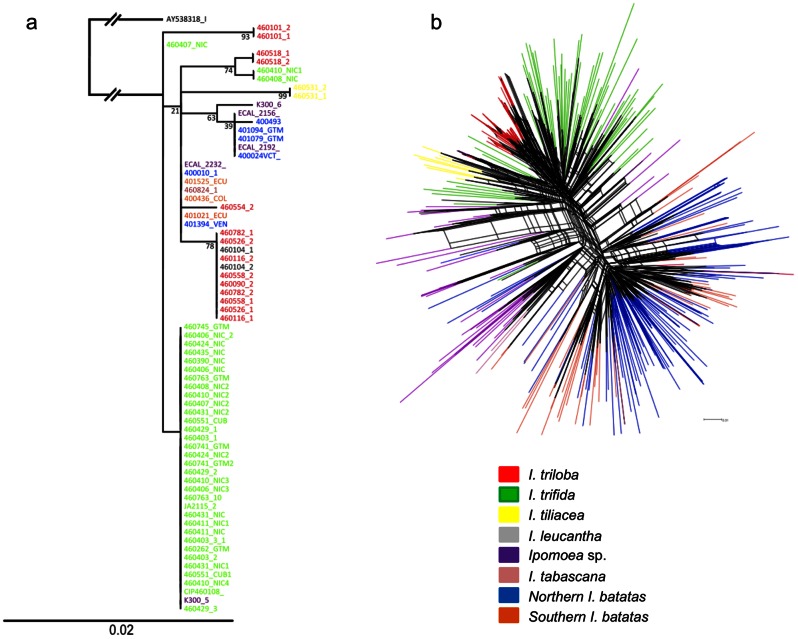
Genetic relationships of *I. batatas*, five wild relatives and *Ipomoea* sp. accessions based on nuclear DNA analyses. a) Maximum likelihood tree based on ITS sequences. Bootstrap values are indicated for central nodes. b) NeighborghNet diagram based on Jaccard distance for nuclear SSR data.

Aligned sequences were 701 bp long. Forty-two haplotypes were obtained considering ambiguous characters, and only 11 when excluding these polymorphisms. Maximum likelihood (Figure 3a) and Neighbor joining analysis (Figure S2) resulted in similar topology, both with a relatively poor resolution. Consistent with the findings on cpDNA sequences, *I. batatas* shared no ITS sequences with *I. trifida* nor with *I. triloba*. Both trees showed that haplotypes were mostly grouped by species (excepted a few *I. triloba* and *I. trifida* which probably represent misidentifications or alternatively hybrids)(Figure 3a). The *I. tabascana* and *Ipomoea* sp. accessions shared (or are grouped with) *I. batatas* haplotypes, except for accession K300-5 (sharing its haplotype with most of *I. trifida* accessions). It should also be noted that *I. batatas* haplotypes are distributed on two distinct branches in the tree (Figure 3a and S2).

### Interspecific relationships as inferred from SSR markers

SSRs could be amplified for all loci and all species, leading to a total of 137 alleles. The number of alleles *NA*, rarefied allelic richness *Ar*, and expected heterozygosity *He*, were similar in *I. trifida*, *I. batatas* and *Ipomoea* sp. groups (Table 1), which also shared the same alleles. *Ipomoea triloba* contains fewer alleles than the other species (on average 3.8 alleles per locus, as compared to 9.5 to 12.6 alleles per locus for *I. trifida*, *I. batatas* and *Ipomoea* sp.). All diversity indices calculated (*Ar*, *He* and *Ho*) showed the same trend. The low values for both *Ho* and intra-taxon differentiation (*D*) in *I. triloba* suggest the presence of null alleles in this species, all the more so since *I. triloba* alleles were quite different from those amplified for the other species. Although the most frequent alleles were shared among all groups, four “specific” alleles were present at a frequency greater than 0.1 in *I. triloba* and less than 0.02 in the other groups. Intra-taxon differentiation (mean pairwise Lynch distance between genotypes) was lower among *I. batatas* (0.424) than among *Ipomoea* sp. (0.566) or among *I. trifida* (0.716).

**Table 1:** Genetic diversity of the four geographically well-sampled taxa as revealed by nuclear SSRs.

Taxa	*I. batatas* (139)				*I. trifida* (65)				*Ipomoea* sp. (39)				*I. triloba* (26)		
Locus	NA	Ar	He	Ho	NA	Ar	He	Ho	NA	Ar	He	Ho	NA	He	Ho
**J206A**	9	6.5	0.70	0.95	9	7.5	0.81	0.6	7	6.8	0.81	0.83	3	0.58	0.11
**J544b**	8	7.1	0.74	0.97	12	10.4	0.84	0.52	7	6.8	0.72	0.75	5	0.73	0.19
**J263**	7	5	0.73	0.91	7	5.9	0.73	0.35	4	4	0.59	0.70	2	0.18	0.11
**J116A**	15	13	0.83	0.99	10	8.5	0.81	0.61	9	8.8	0.82	0.81	3	0.56	0.04
**IbS11**	13	10	0.83	0.99	15	11.1	0.87	0.66	9	8.8	0.83	0.97	1	0.42	0.16
**IbC5**	13	10	0.80	0.97	12	9.6	0.87	0.66	10	9.9	0.84	0.89	8	0.86	0.12
**ib297**	24	14.9	0.85	0.74	30	20.3	0.97	0.44	18	17.1	0.9	0.64	6	0.79	0.08
**J522A**	10	6.4	0.74	0.94	6	5.4	0.80	0.54	12	11.2	0.76	0.86	2	0.46	0.04
*Mean*	*12.38*	*9.1*	*0.78*	*0.93*	*12.62*	*9.8*	*0.84*	*0.55*	*9.5*	*9.2*	*0.78*	*0.81*	*3.75*	*0.57*	*0.1*
D	0.424				0.716				0.566				0.36		

Values for the number of alleles (*NA*), its rarefied value over 25 individuals (1000 resamplings; *Ar*) and the observed and expected heterozygosities *Ho* and *He*, are provided both per locus and as mean values averaged over all loci. *D* corresponds to the intra-taxon mean Lynch distance between genotypes.

In the NeighborNet diagram (Figure 3b), wild relatives and cultivated *I. batatas* formed well separated clusters. Within the cluster of wild relatives, *I. triloba* and *I. tilicaea* were grouped in two distinct lineages, both nested within *I. trifida* accessions. *I. tabascana* and *Ipomoea* sp. accessions were intermediate between the cultivated and the wild relatives clusters, with a few of them clearly associated to the *I. trifida* group. Southern sweet potato varieties tend to be grouped with each other, as well as did Northern ones. However, considerable overlaps are observable and the genetic distinction between Southern and Northern genepools is not clearly identifiable with this representation.

**Figure 4:**
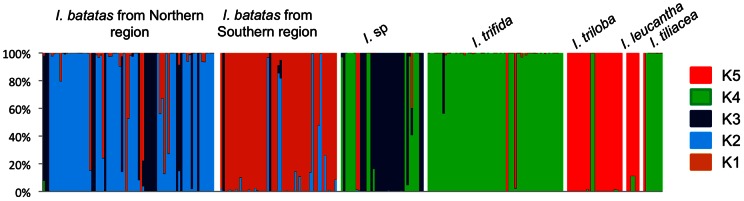
Taxa boundaries as accessed with DAPC analysis for nuclear SSR data. Diagram representing the proportion of membership probabilities in nuclear five clusters (K1, K2, K3, K4 and K5) as determined by the DAPC analysis. Each individual is represented as a vertical bar, with colours corresponding to membership probabilities to the five clusters.

For the DAPC clustering analysis (Figure 4), the appropriate number of clusters was five. This grouping also quite well reflects species boundaries: *I. trifida* accessions are represented by cluster K4 and *I. triloba* accessions by cluster K5. *I. batatas* accessions were associated to three different clusters, K1, K2 and K3. Some *Ipomoea* sp. were attributed to *I. trifida* cluster (K4) and others to the *I. batatas* cluster (K1 and K3; Figure 4). Most of the *I. batatas* accessions from the Southern region (48/56) were grouped in cluster K1 (with one *Ipomoea* sp. from Ecuador and also some *I. batatas* from the Northern region (5/83)). *I. batatas* accessions from the Northern region were subdivided in two clusters, cluster K2 including a large part of these Northern accessions (50/83) and cluster K3 including some accessions from the Northern region (19/83) and some *Ipomoea* sp. (23/42).

With the model-based clustering analysis (Structure, Figure S3), the optimal number of clusters to describe the data was unclear. Consequently, clustering results were less informative (taxon boundaries were not clearly identifiable and many individuals had a mixed genetic constitution; Figure S2). The best Bayesian grouping to be compared with DAPC results was obtained for K = 6, a clustering solution which distinguished cultivated *I. batatas* accessions from wild relatives, and also separated varieties from the Northern and Southern region (Figure S3).

### Congruence between cpDNA haplotype groups and nuclear SSR genetic structure

**Figure 5:**
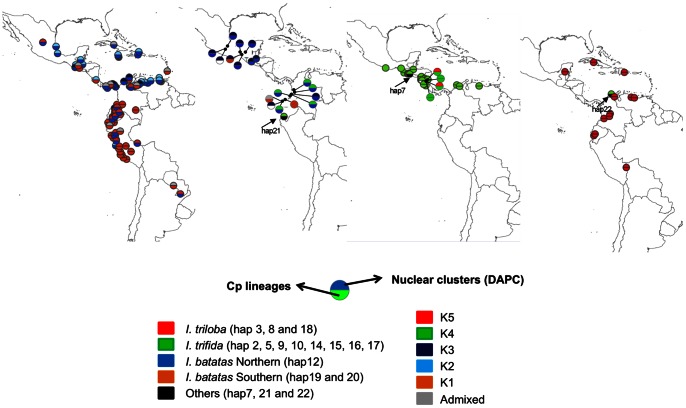
Geographical patterns of cpDNA lineages and nuclear clusters (DAPC results) of *I. batatas*, *I. trifida, I. triloba* and polyploid *Ipomoea* sp. accessions. The bottom half of the circle provides the chloroplast lineage while the top half gives the nuclear genome as revealed by DAPC grouping. When the membership probability to a given cluster is less than 0.8, the accession was considered as admixed. Each circle represents one accession, unless samples of the same accession provided different information. In this case, all combinations encountered are provided. They appeared connected to a black point which indicates their locality.

Both kinds of markers identified diploid *I. trifida* and *I. triloba* as two distinct and uniform genetic groups (Figure 5 and Table 2). Concerning *I. batatas*, we did not sequence all the 139 varieties for the rpl32-trnL(UAG) marker. Thus, we used cpDNA lineage information from Roullier *et al*. [29] to complete our dataset. As described in Roullier *et al*. [29], i) nuclear markers reflect a stronger phylogeographic signal than chloroplast markers but ii) phylogeographic patterns revealed by both sets of data were globally congruent. Indeed, Southern varieties were mostly associated to chloroplast lineage 1 and nuclear cluster 1 (39/54 in total). In the Northern region, both signals were also congruent since 43/84 sweet potato accessions were associated to nuclear clusters K2 and K3 and chloroplast lineage 2. However, 23 Northern varieties were associated to nuclear clusters K2 and K3, yet carried a chloroplast lineage1 haplotype. *Ipomoea* sp. specimens that grouped with the *I. trifida* cluster K2 harbored the Northern chloroplast haplotype (or the unclassified rare haplotype 1) and were all located in the Southern region (Ecuador and South Colombia). Those from the Northern region carried the Northern chloroplast haplotype and were grouped with nuclear cluster K3 (Figure 5 and Table 2).

**Table 2:** Contingency table comparing cpDNA haplotype “lineages” with DAPC clusters among the different taxa *I. batatas*, *I. trifida*, *I. triloba*, *Ipomoea* sp. (including *I. tabascana*).

	Cp lineage 1			Cp lineage 2			*I. trifida* haplotypes	*I. triloba* haplotypes
	Southern *I. batatas*	Northern *I. batatas*	*Ipomoea* sp.	Southern *I. batatas*	Northern *I. batatas*	*Ipomoea* sp.	*I. trifida*	*I. triloba*
**K1**	39	6	3	9	7		1	
**K2**	1	22		4	26			
**K3**	1	1	2		17	10		
**K4**						9	60	
**K5**							2	24
**Admixed**	2	5	1		3		1	

*I. trifida* haplotypes correspond to hap2, 5, 9, 10, 14, 15, 16, 17 and *I. triloba* haplotypes to hap3, 8 and 18.

## Discussion

### The botanical origin of *Ipomoea batatas*

Two scenarios were previously proposed for the formation of the *I. batatas* polyploid genome: autopolyploidization from *I. trifida* [26], or allopolyploidization involving two distant species [25]. The autopolyploidization scenario assumes *I. trifida* to represent an autopolyploid complex, with different ploidy levels (from diploid to hexaploid) from which cultivated *I. batatas* derived. However, cytological and marker-based studies suggested that the *I. batatas* hexaploid genome may be composed of two closely related genomes and a third one from a more distant relative [19], [44]. Austin [25] postulated that the wild ancestor of *I. batatas* was a hybrid between *I. trifida* and *I. triloba* (allopolyploid scenario).

#### Which progenitors were involved?

The data obtained for the different markers identified *I. trifida* as more closely related to sweet potato than *I. triloba,* thereby confirming previous results [20]-[24]. Based on monoparentally inherited chloroplast sequences, *I. trifida* and *I. batatas*, together with *Ipomoea* sp., form a strongly supported monophyletic clade, demonstrating that the maternal genomes that have contributed to the hexaploid *I. batatas* genome are closely related to *I. trifida*. Both the low number of nuclear SSR alleles encountered for *I. triloba* and their strong deficit of heterozygotes suggest that null alleles were frequent in this species. On the contrary, *I. trifida* and *I. batatas* exhibited a quite similar overall allelic composition. The low transferability of SSR markers between *I. batatas* and *I. triloba* (in contrast to *I. trifida*) indicates that *I. triloba* is more distantly related to sweet potato than is *I. trifida*. Interestingly, such genetic relationships were not clearly apparent on the ITS trees, which exhibited a quite poor resolution. Furthermore, neither the NeighborNet diagram, the DAPC analysis, nor the ITS trees, provided any evidence for an interspecific hybrid origin of the sweet potato, and in particular for the involvement of *I. triloba*. Indeed, grouping obtained with these different approaches reflected well taxon boundaries. This absence of evidence for an allopolyploid origin led us to turn to the autopolyploid scenario. However, the absence of shared haplotypes for nuclear and chloroplast sequences between *I. trifida* and any *I. batatas* gene pool further suggests that diploid *I. trifida* thus cannot be considered as the direct wild progenitor of cultivated *I. batatas*. The closest wild relatives of domesticated sweet potato may instead be found among the polyploid accessions of uncertain taxonomic status (*Ipomoea* sp.).

#### Multiple origins

Two distinct *I. batatas* chloroplast lineages were identified. Both share ancestry with *I. trifida*, but they show more divergence among each other than each does with *I. trifida.* This result points towards at least a double origin of *I. batatas* from polymorphic or divergent populations of its progenitor. Multiple origins are also suggested by the DAPC analysis and the NeighborNet diagram. As previously identified [29], both analyses confirmed the existence of two distinct sweet potato nuclear genepools in tropical America, globally consistent with chloroplast lineages and quite geographically restricted.

**Figure 6:**
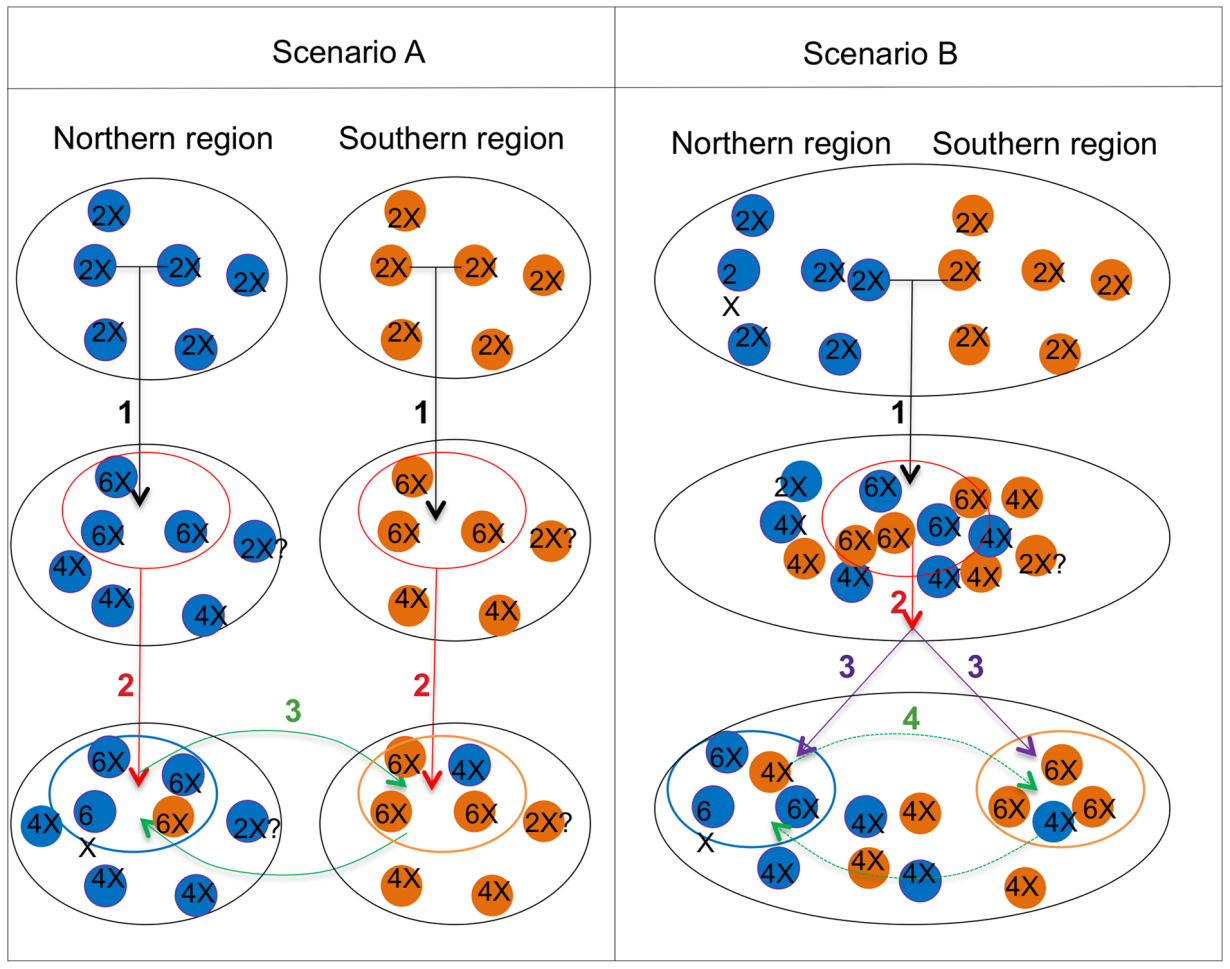
Two possible scenarios about the origins of *Ipomoea batatas.* a) Scenario A which represents according to us, the most parsimonious scenario explaining the clear-cut phylogeographical pattern inferred from both nuclear and chloroplast data: 1) Multiple independent events of autopolypoidy within several polymorphic and pre-differentiated wild populations (phylogeographical differentiation), and then 2) multi-local domestication within each polyploid population, followed by 3) gene flow between the two cultivated genepools and between cultivated and wild forms. b) Scenario B: 1) Hybridization between differentiated conspecific wild populations (in contact because of potential climate-induced or human-induced range shift) and polyploidization, followed by 2) the domestication of these polyploids forms and then 3) patterns of post-domestication human expansion may have been responsible for the clear-cut phylogeographical pattern found within cultivated *I. batatas* in tropical America. Finally, 4) Gene flow between the two cultivated genepools and between cultivated and wild forms may also have occurred.

Autopolyploidy has traditionally been considered to be the duplication of very similar genomes. It is now appreciated that multiple origin of autopolyploidy is common [7], [45], [46]. Autopolyploid complexes may evolve by multiple independent genome duplication events [45], or alternatively result from the hybridization and genome doubling of differentiated con-specific populations brought into contact, for example, by climate-induced range shifts [46]. Such “intermediary” cases are sometimes referred to as segmental allopolyploids, where the genomes involved are sufficiently similar to form multivalents in meiosis [47], but progenitors still considered as distinct species. Both scenarios should be considered for the origin of the “autopolyploid” *I. batatas* complex. However, present data do not allow us to discriminate between them (Figure 6).

#### Markers limitations for resolving the allopolyploid versus autopolyploid origin of the sweet potato

Markers used in the present study are limited and prevent us from firmly reaching the conclusion that *I. batatas* is an autopolyploid. First, as plastid data are usually maternally inherited in angiosperms [48], they only inform on maternal contributors. The level of variation of nuclear ITS markers was low, even among clearly different species. These markers do not appear informative enough for resolving genetic relationships at this level. In addition, no cloning was performed in our study, thus not all potential homologous sequences could be read, giving again only a partial picture of the phylogenetic relationships between polyploidy species. Moreover, ITS loci may be affected by concerted evolution, which can homogenize the sequences even across homologous loci, essentially obscuring evidences of the contribution of one or more ancestral genomes [28]. Therefore, a polyploid may or may not have conserved ITS sequences from all of its ancestors. Our SSRs markers also have some limitations. First, they were not designed to distinguish among different genomes present in the sweet potato, and the scoring used here did not allow determining their copy number. They provide an overall assessment of similarity between genomes which do not necessarily reflect phylogenetic relationships among these species. Also, it is quite unclear how genetic analysis such as DAPC or distance-based tree are appropriate for treating microsatellites data in a polyploid context, especially when data on microsatellite alleles were converted to binary data and when multiple ploidy levels are compared. Such analyses may result in the clustering of taxa with the same ploidy level regardless of their true genetic relationships.

To obtain further insights into where, how, and when polyploidization and sweet potato domestication occurred, marker-based studies should focus on gene trees reconstruction and consider both homologous (similar gene copies within a progenitor diploid genome) and homologous (similar gene copies brought together in a polyploid genome after the hybridization and genome doubling of differentiated diploid progenitors) copies of several independent single-copy (per genome) nuclear genes [49].

### Samples of uncertain taxonomical status and the origin of sweet potato

At least two polyploidization/hybridization events, implying the existence of triploid or tetraploid intermediates, are necessary to explain how hexaploid sweet potato could have been generated from diploid progenitors (Figure S4). Kobayashi [26] proposed that these intermediates might resemble the wild polyploids that have been collected from Mexico to northern Peru, accessions first identified as *I. trifida*. Taxonomical revision subsequently placed most of them in *I. batatas* [27]. This latter re-identification was supported by the fact that these wild *Ipomoea sp*. accessions were more easily crossed with *I. batatas* than with diploid *I. trifida* [50].

Among the polyploid *Ipomoea* sp. accessions used in our study, numerous ones shared haplotypes with *I. batatas*, but none with *I. trifida*. The few *Ipomoea sp.* accessions collected in Ecuador carried a Southern *I. batatas* chloroplast haplotype, while all other *Ipomoea* sp. accessions carried the Northern *I. batatas* chloroplast haplotype. Nuclear SSRs placed *Ipomoea* sp. accessions as intermediate between diploid *I. trifida* and cultivated *I. batatas*. While four accessions (seven samples) of *Ipomoea* sp., all from Colombia, were clearly assigned to the diploid *I. trifida* cluster K2, all others grouped with *I. batatas* Northern cluster K3. Like most *Ipomoea* sp. accessions, *I. tabascana*, the putative hybrid between *I. batatas* and *I. trifida,* carried a nuclear genome attributed to the cluster K3 and a Northern *I. batatas* chloropalst haplotype. These specimens may represent original wild *I. batatas*, i.e., forms intermediate between the diploid progenitors and cultivated hexaploid *I. batatas* (as was strongly suggested by Austin [25]; and Bohac *et al.* [27]. Alternatively, they may be feral individuals or even hybridized forms issued from crosses between cultivated hexaploid *I. batatas* (as the maternal parent) and diploid *I. trifida* (Although mainly clonally propagated by farmers, hexaploid *I. batatas* is still able to reproduce sexually - crosses between 6X sweet potatoes as well as with *I. batatas* accessions with lower ploïdy levels are allowed - and to hybridize with diploid *I. trifida* [50]). Current genetic data are not sufficient to clarify their status. However our genetic results together with previous taxonomic studies, which identify polyploid (3X, 4X and few 6X) *Ipomoea* sp. in the *I. batatas* species, are additional evidence that *I. batatas* may exist not only as hexaploid cultigens, but also as a true wild species, with several ploidy levels (from 3X to 6X at least), forming in fact a complex with multiple origins.

Recently, an analysis of *Waxy* intron variations argued for an allopolyploid origin for the sweet potato, which probably occurred by hybridizations between *I. tenuissima* and *I. littoralis* [51]. *I littoralis* was described by Austin [52] to be the only species of the genus native and endemic to the Old World. Some wild tropical American tetraploid plants may have been misidentified as *I. littoralis* (Kobayashi, [26]). *I. littoralis* accessions used in the study by Gao *et al*. [51] likely indeed are specimens of wild *I. batatas*. Their results would then confirm ours in supporting the fact that *I. batatas* has multiple origins. Further elucidation of the evolutionary history of cultivated *I. batatas* will require a better understanding of the distribution and ecology of these wild, cultivated and intermediate forms of *I. batatas* throughout the Neotropics, sampling and considering them in future genetic studies.

### How, when and where was sweet potato domesticated?

It is quite clear that crop origins in Neotropical America were spatially diffuse, and often occurred in a number of localities in both tropical Central and South America [15]. Previous molecular analyses suggested that sweet potato may be Central American in origin [53]. Results of a recent study [29] suggest that at least two domestications occurred, one in Caribbean/Central America, and one in northwestern South America, giving rise to two domesticated genepools (the Northern and Southern ones). Our study confirms this phylogeographic pattern, but does not allow us to pinpoint where domestication took place, because we still lack a representative sample of wild *I. batatas* populations. In one scenario (scenario A, Figure 6), the *I. batatas* complex may have evolved in distinct geographical areas (perhaps at the periphery of ranges of their diploid ancestors), raising the possibility that two independent groups of cultivators took advantage of distinct local wild polyploid *I. batatas* populations. Following this scenario, these two gene pools have secondarily come into contact, as shown by their admixture for both chloroplast and nuclear markers (Figure 5). Alternatively, the phylogeographic pattern found within *I. batatas* might result from post-domestication patterns of human expansion (Figure 6, scenario B). In scenario B, a single domestication from polymorphic wild polyploid populations (resulting from independent, geographically restricted autopolyploidization events or from the contact, hybridization and genome doubling of pre-differentiated populations) may have occurred. Considering all available evidence, scenario A seems the most parsimonious. Moreover, other scenarios involving more complex temporal relationships between polyploidization and domestication could also be considered. Following a recent study on the Mimosoid legume tree *Leucaena*, human predomestication cultivation activities, by putting in artificial sympatry different *Leucaena* species, may have favored their hybridization and polyploidization, events which consequently constituted “a potent trigger” for the achievement of the domestication process [54].

Sweet potato is one of the oldest domesticates in the Americas, with archaeological remains of dried sweet potatoes from Peru dating back to 10,000 to 8,000 BP [15], [55]. Unlike other plants (maize, wheat or pearl millet, for example), cultivated *I. batatas* has not suffered a severe bottleneck during domestication: the crop's nuclear genetic diversity, at least as estimated based on neutral markers, is comparable to that of its polyploid progenitor, and only slightly lower than that of diploid *I. trifida* in terms of number of alleles. Autopolyploid formation allows a large part of the genetic diversity present in progenitors to be incorporated, particularly when multiple maternal lineages are added [7]. However, it is also possible that the domestication process in itself resulted in retention of a large part of the diversity of ancestral wild populations. The major trait of sweet potato's domestication syndrome is the development of edible tuberous storage roots, a trait with complex determinism [56], likely linked to polyploidy. Indeed, diploid *I. trifida* do not produce edible roots, but often present some thickened ones. Wild tetraploid *I. batatas* form only thickened “pencil-shaped” roots [27] and wild hexaploid *I. batatas* populations have been too scantily characterized to provide any reliable description of their roots. Even in the cultivated forms, tuberization is a labile trait, with complex genetic and environmental determinism and low heritability [50], [57]. Moreover, gene flow between the crop and wild relatives (*I. trifida*, and more particularly wild *I. batatas*) is still possible in natural settings despite their different ploidy levels, but artificial crosses show reduced seed set and poor yields [50], [58]. With current data, it is not possible to establish a tight framing of the timing of domestication relative to polyploidization. We postulate, however, that during the domestication process, cultivators may have repeatedly captured and multiplied wild mutants with tuberous roots, probably hexaploid plants. Crop/wild gene flow would have progressively decreased under cultivation, favouring recombination between tuber-bearing cultivated forms and stabilizing the formation and development of storage roots. The domestication of clonally propagated crops is not an instantaneous event, i.e. capture and multiplication of genotypes with desirable traits. As for seeds propagated crops, a succession of “recombination-and-selection” cycles has been necessary to assemble traits of the domestication syndrome [59].

## Conclusions

Previous cytogenetic and neutral-marker-based studies have pointed to diploid *I. trifida* as the closest wild relative of sweet potato. We argue here that polyploid true wild *I. batatas* populations exist. Diploid specimens similar to their diploid progenitors have yet to be identified. Sharing ancestry with extant diploid *I. trifida*, these putative diploids might be extinct or may simply have not been collected. We proposed that wild tuber-bearing populations may have been domesticated independently in South America and the Caribbean/Central America, two gene pools that have secondarily come into contact along human movements.

## Acknowledgments

The authors are very grateful to D.F. Austin (Arizona-Sonora Desert Museum) for useful comments on previous versions of the manuscript and for stimulating discussions. We thank E. Emschwiller, S. Joly, and an anonymous referee for their comments on an earlier draft, and CIP field technicians for providing plant material. Data used in this work were produced through the technical facilities of the Centre Méditerranéen Environnement Biodiversité.

## Supporting Information

Figure S1 **IGS haplotypes majority rule consensus tree obtained with Bayesian and Maximum-likelihood reconstruction methods.** Numbers along branches indicate bayesian posterior probabilities (first value) and bootstrap values (second value). Branches are colored according to the species they represent. When several species contained the same haplotype and were grouped on the same branch, dashed with the different corresponding colors were layed on the tree branches.
(TIFF)

Figure S2 **Neighbour-joining tree based on Hamming distance between ITS haplotypes.** Bootstrap values >50 are indicated for central nodes. Accessions names are those referenced in the Table S1.
(TIFF)

Figure S3 **Results obtained with the Bayesian clustering method.** a) Variation of the posterior log-probability of the data as a function of the number of clusters. Values of likelihood increased from K = 1 to K = 10, showing that the fit of the model to the data is continuously improved when the number of clusters is increased. b) Variation of ΔK values. The optimal number of clusters to describe the data was unclear. c) Proportion of ancestry shared within each cluster for K = 5 and K = 6. Each individual is represented as a vertical bar, with colours corresponding to probabilities of assignment to the different clusters. For comparison purposes, individuals order in the diagram is the same than that used in the Figure 4.
(TIF)

Figure S4 **Mechanisms of formation of the hexaploid genome of sweet potato.** The formation of the hexaploid genome must have involved at least two steps, from diploidy to intermediate ploidy levels (triploid or tetraploid) and then hexaploidy. The most likely polyploidization route in sweet potato involves sexual mechanisms via the production of 2 n gametes, whose occurrence has been demonstrated in diploid and triploid *I. trifida*, as well as in tetraploid *I. batatas*. Morever, polyploid *Ipomoea* sp. (mostly 4X) analyzed in our study may have two distinct origins: i) original intermediate wild forms of *I. batatas* (solid circle), or ii) feral plants issued from crosses between hexaploid I. batatas and a diploid wild relative (same but transparent colored circles).
(PDF)

Table S1 **Passport data of the accessions used in the present study.** Taxa name, ploidy level, *ex situ* collection names (CIP, NIAS, USDA), geographical origin data and GenBank.
(XLS)

Table S2 **Summary of the number of accessions typed for the three kinds of markers (-IGS- chloroplast sequence, -ITS- nuclear sequence and nuclear microsatellites –SSR-) and their geographic origins.** For wild Ipomoea species, accessions refer to a population; the number of samples used is indicated between brackets. Accessions from the northern, Caribbean part of Colombia were attributed to the Northern region, while those from southern Colombia were placed in the Southern region (Figure 1).
(XLS)
