# Correction: Disentangling the Origins of Cultivated Sweet Potato (*Ipomoea batatas* (L.) Lam.)

**DOI:** 10.1371/annotation/936fe9b4-41cb-494d-87a3-a6d9a37c6c68

**Published:** 2013-10-15

**Authors:** Caroline Roullier, Anne Duputié, Paul Wennekes, Laure Benoit, Víctor Manuel Fernández Bringas, Genoveva Rossel, David Tay, Doyle McKey, Vincent Lebot

The last author (VL) should be noted as one of the authors who wrote the paper.

