# Supplementary figures and images for: Disentangling the Origins of Cultivated Sweet Potato (Ipomoea batatas (L.) Lam.)
Source: PLoS One. 2013 May 27;8(5):e62707. doi: 10.1371/journal.pone.0062707 (PMC3664560; doi:10.1371/journal.pone.0062707)

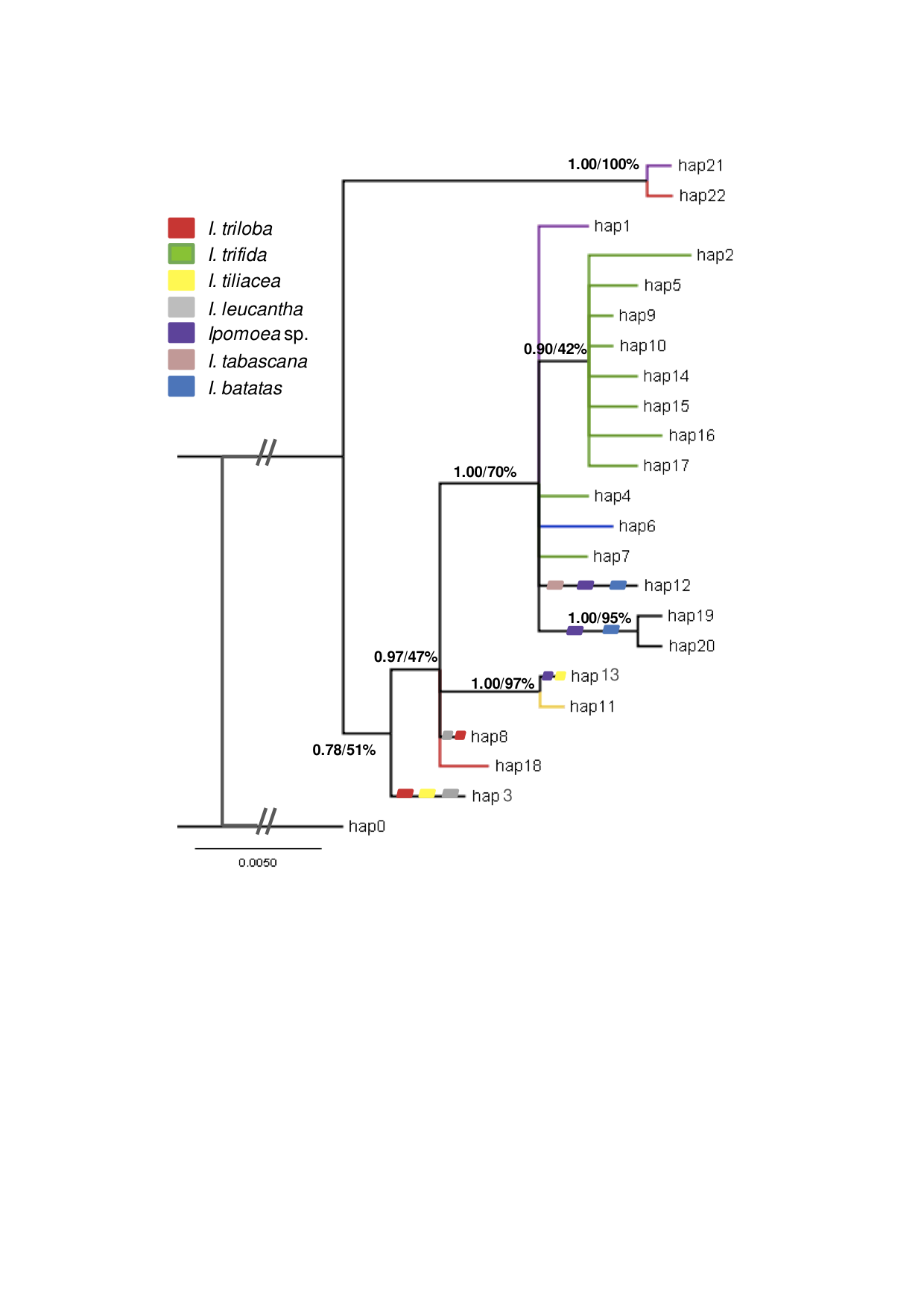

Supplement: Figure S1 — IGS haplotypes majority rule consensus tree obtained with Bayesian and Maximum-likelihood reconstruction methods. Numbers along branches indicate bayesian posterior probabilities (first value) and bootstrap values (second value). Branches are colored according to the species they represent. When several species contained the same haplotype and were grouped on the same branch, dashed with the different corresponding colors were layed on the tree branches. (TIFF) [file pone.0062707.s001.tiff]

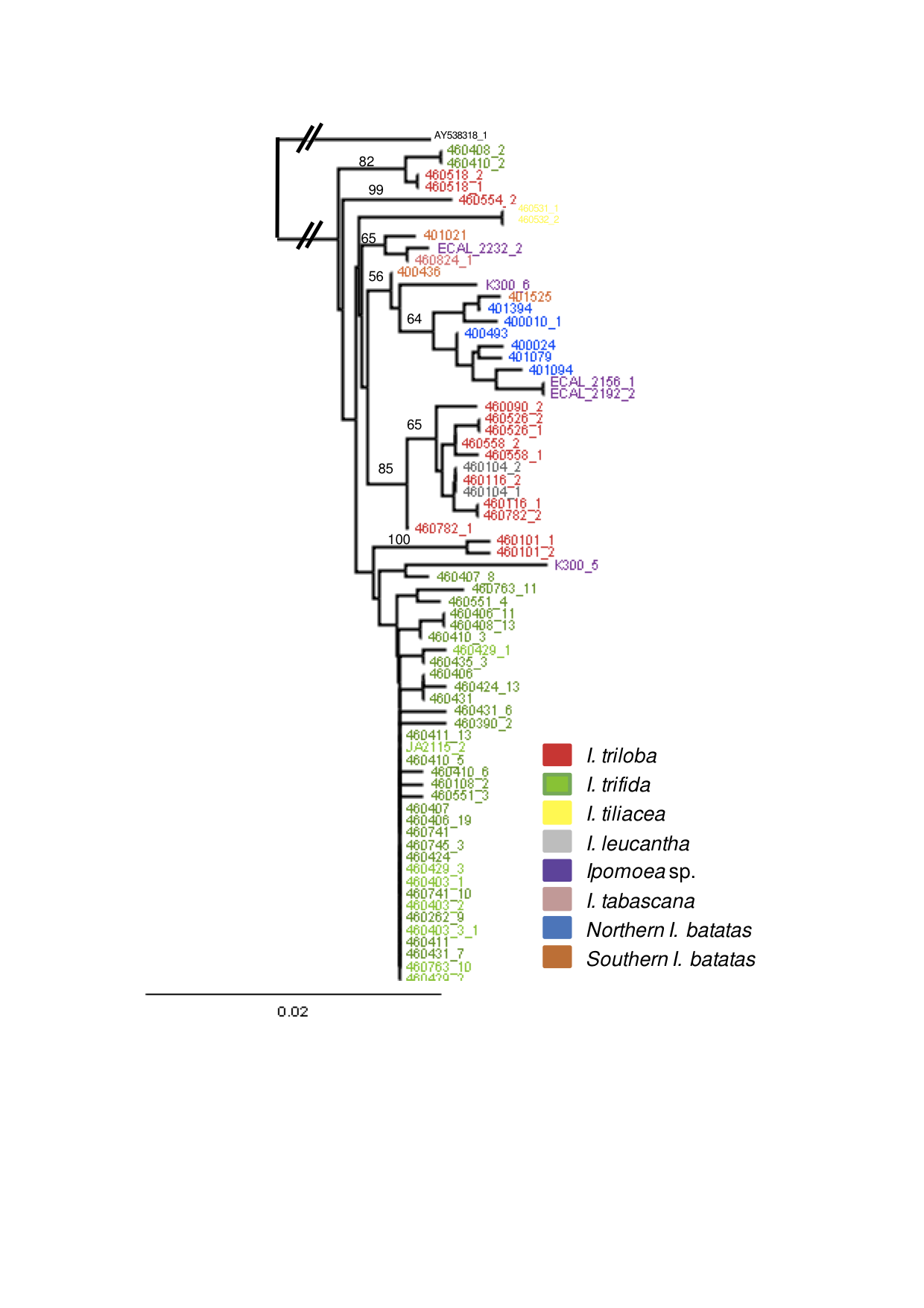

Supplement: Figure S2 — Neighbour-joining tree based on Hamming distance between ITS haplotypes. Bootstrap values >50 are indicated for central nodes. Accessions names are those referenced in the Table S1. (TIFF) [file pone.0062707.s002.tiff]

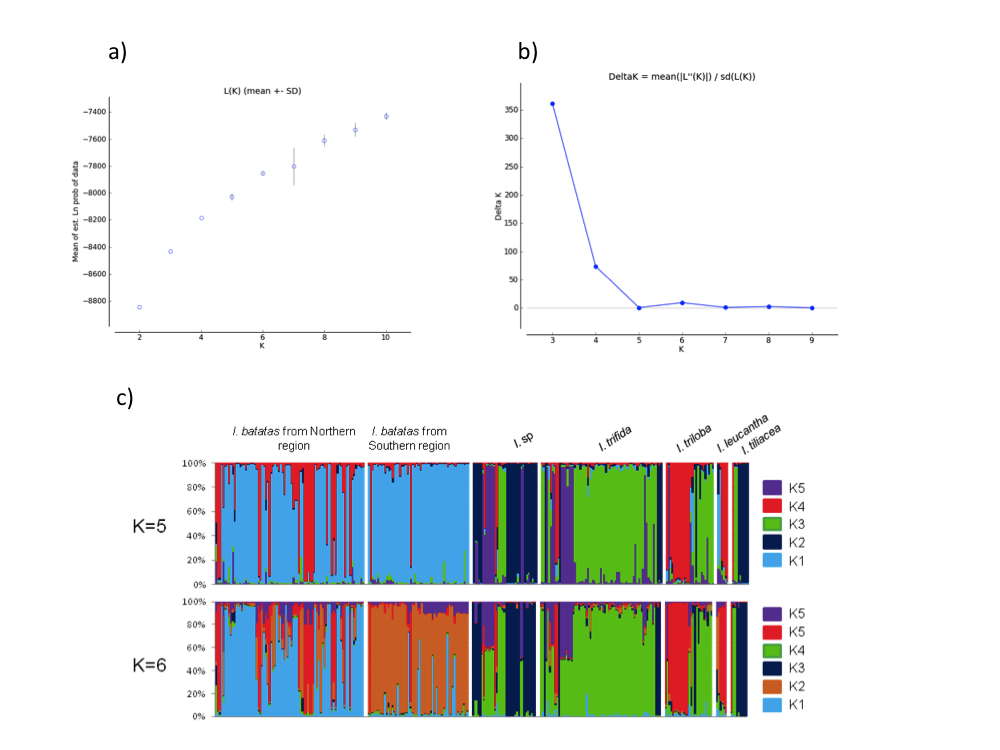

Supplement: Figure S3 — Results obtained with the Bayesian clustering method. a) Variation of the posterior log-probability of the data as a function of the number of clusters. Values of likelihood increased from K = 1 to K = 10, showing that the fit of the model to the data is continuously improved when the number of clusters is increased. b) Variation of ΔK values. The optimal number of clusters to describe the data was unclear. c) Proportion of ancestry shared within each cluster for K = 5 and K = 6. Each individual is represented as a vertical bar, with colours corresponding to probabilities of assignment to the different clusters. For comparison purposes, individuals order in the diagram is the same than that used in the Figure 4. (TIF) [file pone.0062707.s003.tif]
