# Supplementary material for: Disentangling the Origins of Cultivated Sweet Potato (Ipomoea batatas (L.) Lam.)
Source: PLoS One. 2013 May 27;8(5):e62707. doi: 10.1371/journal.pone.0062707 (PMC3664560; doi:10.1371/journal.pone.0062707)
Supplement: Figure S4 — Mechanisms of formation of the hexaploid genome of sweet potato. The formation of the hexaploid genome must have involved at least two steps, from diploidy to intermediate ploidy levels (triploid or tetraploid) and then hexaploidy. The most likely polyploidization route in sweet potato involves sexual mechanisms via the production of 2 n gametes, whose occurrence has been demonstrated in diploid and triploid I. trifida, as well as in tetraploid I. batatas. Morever, polyploid Ipomoea sp. (mostly 4X) analyzed in our study may have two distinct origins: i) original intermediate wild forms of I. batatas (solid circle), or ii) feral plants issued from crosses between hexaploid I. batatas and a diploid wild relative (same but transparent colored circles). (PDF) [file pone.0062707.s004.pdf]

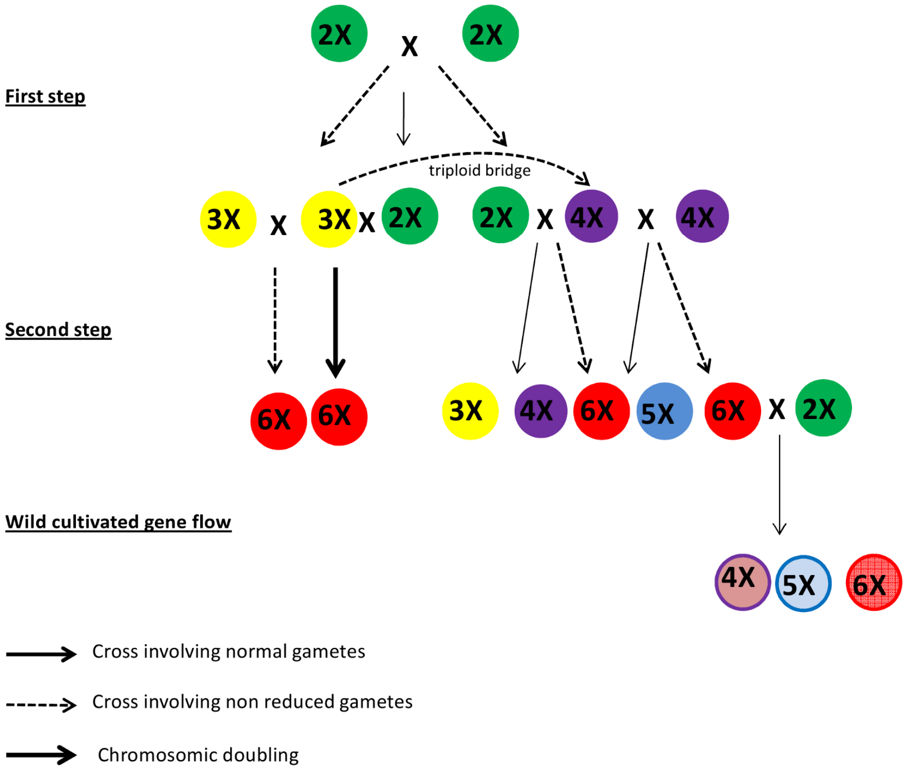

Triploid bridge: formation of triploids is often considered to mediate the autopolyploidization process. It is also possible that tetraploids, as well as hexaploids, were formed without the intermediate formation of triploids, simply via successive crosses involving 2n gametes (right part of the graph).
